# The Relevance of Heart Rate Fluctuation When Evaluating Atrial Substrate Electrical Features in Catheter Ablation of Paroxysmal Atrial Fibrillation

**DOI:** 10.3390/jcdd9060176

**Published:** 2022-06-01

**Authors:** Aikaterini Vraka, José Moreno-Arribas, Juan M. Gracia-Baena, Fernando Hornero, Raúl Alcaraz, José J. Rieta

**Affiliations:** 1 BioMIT.org, Electronic Engineering Department, Universitat Politecnica de Valencia, 46022 Valencia, Spain; aivra@upv.es; 2 Cardiology Department, Saint John’s University Hospital, 03550 Alicante, Spain; jomoreno@gmail.com; 3 Cardiovascular Surgery Department, Hospital Clínico Universitario de Valencia, 46010 Valencia, Spain; gracia_juabae@gva.es (J.M.G.-B.); hornero_fer@gva.es (F.H.); 4 Research Group in Electronic, Biomedical and Telecommunication Engineering, University of Castilla-La Mancha, 16071 Cuenca, Spain; raul.alcaraz@uclm.es

**Keywords:** atrial fibrillation, catheter ablation, coronary sinus, P-waves, correlation, atrial substrate, invasive recordings, heart-rate adjustment

## Abstract

Coronary sinus (CS) catheterization is critical during catheter ablation (CA) of atrial fibrillation (AF). However, the association of CS electrical activity with atrial substrate modification has been barely investigated and mostly limited to analyses during AF. In sinus rhythm (SR), atrial substrate modification is principally assessed at a global level through P-wave analysis. Cross-correlating CS electrograms (EGMs) and P-waves’ features could potentiate the understanding of AF mechanisms. Five-minute surface lead II and bipolar CS recordings before, during, and after CA were acquired from 40 paroxysmal AF patients. Features related to duration, amplitude, and heart-rate variability of atrial activations were evaluated. Heart-rate adjustment (HRA) was applied. Correlations between each P-wave and CS local activation wave (LAW) feature were computed with cross-quadratic sample entropy (CQSE), Pearson correlation (PC), and linear regression (LR) with 10-fold cross-validation. The effect of CA between different ablation steps was compared with PC. Linear correlations: poor to mediocre before HRA for analysis at each P-wave/LAW (PC: max. +18.36%, *p* = 0.0017, LR: max. +5.33%, *p* = 0.0002) and comparison between two ablation steps (max. +54.07%, *p* = 0.0205). HRA significantly enhanced these relationships, especially in duration (P-wave/LAW: +43.82% to +69.91%, *p* < 0.0001 for PC and +18.97% to +47.25%, *p* < 0.0001 for LR, CA effect: +53.90% to +85.72%, *p* < 0.0210). CQSE reported negligent correlations (0.6–1.2). Direct analysis of CS features is unreliable to evaluate atrial substrate modification due to CA. HRA substantially solves this problem, potentiating correlation with P-wave features. Hence, its application is highly recommended.

## 1. Introduction

With a prevalence higher than 37 million people worldwide, significant mortality rates, and economic burden, atrial fibrillation (AF) is not only just the most common cardiac arrhythmia, but also a multifactorial threat [1,2]. AF can be classified as a function of its spontaneous termination into two different types: paroxysmal and non-paroxysmal. The former includes AF episodes spontaneously terminating in less than 7 days, while the latter includes AF episodes unable to terminate within 7 days without intervention [2]. Pulmonary veins (PVs) are the principal AF foci, and their catheter ablation (CA) is the cornerstone of AF treatment, especially successful in paroxysmal AF cases [2,3,4,5]. Despite the high success rates of the CA of PVs, AF recurrence is not an unusual phenomenon, especially in persistent AF patients. While early recurrence is mostly associated with PV reconnection, late recurrence may be an indicator of the existence of non-PV triggers [6].

One of the keys to understanding the AF mechanisms and deciding the therapy and personalized follow-up treatment is the detailed analysis of the atrial substrate, which is defined as any changes in structural, electrical, or functional level that sustain the AF activity, also known as atrial remodeling [2]. Although it is not necessary for all of the aforementioned alterations to be present, there is a substantial association between them [7,8,9]. A successful CA outcome involves a sufficient atrial substrate modification, which implies the interception of the mechanisms favoring the AF perpetuation due to the isolation of the PVs. As in the case of atrial remodeling, substrate modification can happen at three levels: structural, electrical, or functional. Since structural changes are connected with the anatomical alteration of the atria, a blanking period of 3 months is typically used in order to safely assess the structural substrate modification [7,10]. Although electrical and functional—related to freedom from AF—modification have been observed as early as one day after CA, electrical substrate modification can in fact be observed even earlier, immediately after the CA procedure [11,12,13].

During CA, one or more atrial structures are chosen as a reference, and recordings from a stationary catheter are acquired throughout the procedure [14,15]. The coronary sinus (CS) is the most common reference, as it forms an interatrial electrical bridge and can initiate or propagate the AF activity [16,17,18,19,20]. CS catheterization allows the mapping of the entire atria. CS pacing can induce AF, and depending on the CS channels where pacing has been performed, triggers in the left (LA) or right atrium (RA), as well as in the CS musculature can be detected [21,22,23]. Nevertheless, variable CS anatomy and vivid cardiac contraction can complicate the CS cannulation procedure, and the selection of the proper CS channel for analysis should be performed with caution and may differ for recordings in AF or in sinus rhythm (SR). Recently, a study alerted the risk of high noise prevalence in recordings from the distal tip of the CS catheter during SR [13].

The pivotal role of CS not only in AF initiation or perpetuation, but also in AF mapping has led to the further investigation into the clinical information that can be acquired from the CS electrograms (EGMs). During AF, the analysis of the dominant frequency (DF) or complexity of CS EGMs from PV and non-PV CA has been quite fruitful in predicting the AF recurrence [24,25,26]. Additionally, DF in CS EGMs has been recruited in order to define the endpoint for the CA of PVs and complex EGMs [27]. The existence of complex EGMs in CS has also been associated with AF prevalence [28]. Despite the high interest in CS EGMs during AF, little or no attention has been paid to CS function during SR and its modification after CA procedures. Prolonged conduction time measured between EGMs from RA and CS in recordings acquired before CA of PVs and complex EGMs has shown high predictive power for atrial tachyarrhythmia occurrence [29]. Moreover, in CA of atrial flutter, fractionation in the EGMs of CS can predict the incidence of AF [30].

Although they present interesting findings, studies focused on the CS analysis during SR are few and mainly involve additional CA applications. Nevertheless, the CS function during CA of PVs only, as well as the alterations that may be provoked by the CA are yet to be discovered. Given the high significance of CS for the CA procedure and the fact that CS EGMs provide information at a local level, an analysis focusing on the CS during the CA of PVs and how the CA procedure modifies its function could potentiate the perception on the AF mechanisms during SR. Any important findings could be recruited along with already established methods in order to provide a new perspective into the AF analysis. The wide availability of CS recordings from CA sessions makes the investigation of this assumption quite feasible. However, the lack of major analyses that could serve as a reference for the observation of the CS function alteration raise the need for alternative studies that are vastly applied to investigate the atrial substrate modification.

During the CA procedure, electrocardiogram (ECG) recordings from the skin surface are simultaneously acquired along with the recordings from the intracardiac catheters and are vastly used to observe the atrial substrate modification. In CA sessions performed under SR, analysis is mainly focused on either of two parameters: P-waves or heart-rate variability (HRV). The P-wave is the ECG part that represents the atrial depolarization [31]. Hence, P-wave variations after CA reflect changes in the atrial contractility. Many studies have revealed the capability of P-wave parameters such as P-wave duration, amplitude, as well as the frequency domain analysis of assessing the CA outcome [32,33,34,35,36,37,38,39]. P-wave duration is probably the most relevant feature, as it measures the duration of the atrial depolarization [40]. The rationale behind P-wave duration analysis is that the P-wave duration values come as a function of the anatomical abnormalities assisting the AF propagation and that the P-wave shortening, especially occurring in the left atrial P-wave part, is indicative in the freedom from AF after the CA procedure [36,38,41,42,43].

Being a measure of calibration between the sympathetic and parasympathetic nervous systems [44], HRV is the other popular evaluator of CA outcome [45,46]. It can be assessed by the variation between successive R-R intervals, high- and low-frequency analysis or Lyapunov exponents and Kolmogorov entropy, among other methods [44,47]. The reduction of HRV after radiofrequency (RF) CA is a natural, albeit temporary, side effect of the CA procedure that is gradually restored over time [48] and can predict AF recurrence [49]. The main mechanisms causing HRV reduction are the RF energy emitted in the PV tissue, stimulating the sympathetic nerve fibers and causing temporal parasympathetic nervous system withdrawal [48]. Recently, it was demonstrated that high parasympathetic tone after cryoballoon CA is also connected with AF recurrence [50].

Despite the high significance of P-waves and HRV analysis for atrial substrate modification evaluation after the CA of PVs, these parameters provide information on the global atrial substrate modification or the ventricular response, in the case of HRV. However, how critical structures are affected by the CA procedure is yet to be discovered. Constant pacing of CS for AF mapping may alter its functionality. Whether and to what extent this is reflected in the conventional substrate modification assessment techniques remain unknown. The aim of the current study is to investigate this presumption. For this purpose, any possible linear or nonlinear correlations between ECG and CS recordings are explored in order to provide new insights into the pathophysiological AF mechanisms during SR and their interaction with the most popular AF treatment, CA.

The remainder is organized as follows. The database and analysis methods are presented in Section 2, where also the statistical analysis is explained. Results are shown in Section 3, and they are further analyzed in Section 4, where relevant work is also described. The manuscript is completed with the conclusions presented in Section 5.

## 2. Methods

Recordings of 40 paroxysmal AF patients undergoing the RFCA of PVs for the first time were employed. Isolation was performed by creating lesions surrounding each PV side (left or right), guided by 3D electroanatomical mapping. The procedure started with the RFCA of the left PVs, followed by the RFCA of the right PVs. Whenever tricuspid isthmus (TCI) block was observed, TCI isolation was performed following RFCA. The endpoint of the procedure was bi-directional electrical isolation of all pulmonary veins after adenosine administration. Recordings from a standard 12-lead electrocardiogram (ECG) and a decapolar CS catheter with a sampling frequency of 1 kHz were acquired by a Labsystem™ PRO EP recording system (Boston Scientific, Marlborough, MA, USA) for five minutes before, during, and after the CA procedure. Recordings during the CA procedure were acquired after the isolation of left pulmonary veins (LPVI). P-wave analysis was performed using recordings from lead II, while the channel of bipolar CS recordings with the least fluctuations and the highest amplitude was selected for the CS analysis. This channel varied among patients, but was always the same for one patient at all three time points from which the recordings were obtained. In case all channels showed a high amplitude and clear signals, medial or mid-proximal channels were selected [13].

### 2.1. Preprocessing

ECG preprocessing contained the removal of power-line interference, high-frequency muscle noise, and baseline wander [51,52]. Ectopic beats were present in some of the recordings, with a maximum prevalence of 4% of total beats. Ectopic beat removal contained the ectopic detection, cancellation, and replacement by linear interpolation [53,54]. After ectopics removal, P-waves were detected and delineated [55,56].

CS recordings’ preprocessing started with denoising, mean removal, and cancellation of ventricular activity, if present, by an adaptive cancellation method [57,58]. Afterwards, local activation waves (LAWs) were detected and delineated [59,60]. Delineation was then inspected and corrected by two experts for both invasive and surface recordings.

### 2.2. Data Analysis

After P-wave and LAW detection, the following features were calculated at each activation for both P-waves and LAWs and then averaged over each recording:

- *Duration:* Once delineated, the interval between the onset and offset of each activation was considered as its duration.
- *Amplitude:* Maximum ($Amp_{max}$) and peak-to-peak (*PP*) amplitudes. For ECGs, P-wave $Amp_{max}$ and *PP* concur, since P-waves in lead II are positive. The root mean square (*RMS*) is the quadratic mean of the function that defines each activation.
- *Area:* Area of the positive parts of the signal (*PosAr*), calculated by the integration over the time interval of the amplitude of each activation with the trapezoidal method.
- *Slope rate:* Increasing or decreasing rhythm at 5%,10%,20% of total duration of each P-wave/LAW, as well as at its maximum point, calculated as:
  (1) $$S_{i}=\frac{Amp(i)-Amp(onset)}{t_{i}-t_{onset}},$$
  where Amp(i) is the amplitude at i=5,10,20% or the peak of the activation, Amp(onset) is the amplitude at the onset, and $t_{i}$ and $t_{onset}$ are the sample points at i=5,10,20% or the peak and the onset, respectively. *Slope rate* is always positive for P-waves, as their peaks always present a positive amplitude. For LAWs, *slope rate* can be negative as well.

Since the analysis of the present study is focused on atrial activations, HRV analysis was calculated by measuring successive P-wave or LAW intervals. Hence, it will be referred to in the remainder of the manuscript as atrial rate variability (ARV). ARV was calculated across each surface and invasive recording analyzing the standard deviation of the normal-to-normal P-wave or LAW interval (*SDNN*), the variance of normal-to-normal P-wave or LAW interval (*VARNN*), and the RMS of successive differences (*RMSSD*) between two P-waves or LAWs [44].

In order to compensate for the effect of fluctuations in heart rate (HR) on several features [61], an HR adjustment (HRA) was performed by scaling them by the following factor:

(2) $$sf_{i}=\frac{1000}{IBI_{i}},$$

where $IBI_{i}$ is the inter-beat interval between the $i^{th}$ and the ${\left(i-1\right)}^{th}$ activations. HRA was used to scale *Duration* (HRA(Duration)) and *Area* (HRA(PosAr)) and scale inversely *Slope rate* ($\mathrm{HRA}(S_{i}$)).

### 2.3. Statistical Analysis

**Figure 1:**
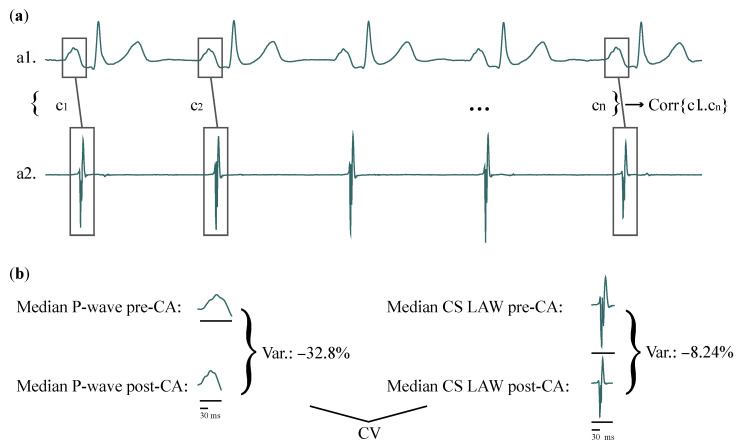
(**a**) Representation of how correlations between features of each P-wave (a1) and CS LAW (a2) of one patient are computed. (**b**) CV of the same patient, example shown for *Duration*, for recordings before and after CA. Variation due to CA in *Duration* is calculated for surface (**left**) and invasive (**right**) recordings. Correlation between the surface and invasive variations is then calculated across the entire patient cohort. CS: coronary sinus; LAW: local activation wave; CV: correlation of variation; CA: catheter ablation.

Any possible linear or nonlinear relationships between P-waves and CS LAWs were investigated. Linear correlations between each P-wave and CS LAW of every recording were assessed with Pearson’s correlation and linear regression with 10-fold cross-validation, and nonlinear relationships were assessed with cross-quadratic sample entropy (CQSE). An example of how linear correlations between each P-wave and CS LAW were calculated can be seen in Figure 1a. As ARV features are calculated across each recording and do not correspond to activation-based analysis, they were excluded from this step.

Correlation of CA-induced variation (CV) between P-wave and CS LAW features including ARV was investigated by Pearson’s correlation, which performs a bidirectional comparison. This step allows the comparison between the effect that CA had on CS function and the effect that CA had on the entire atria. For each patient and each feature, the CA-induced variation was the percentage of alteration, calculated as the median value after CA with respect to the median value before CA:

(3) $$\mathrm{CV}=\left(\frac{value\_\;after}{value\_\;before}-1\right)\times 100\;\left(\%\right).$$

Figure 1b shows an example of how CV is calculated. The normality of the results was tested with the Shapiro–Wilk test, and final correlations are expressed as medians, as the values did not follow a normal distribution [62].

Linear regression can only assess the one-way relationship between a dependent variable *a* and one or more independent variables $b_{1}\;,b_{2},\cdots ,\;b_{n}$, where *n* is the number of independent variables or predictors. Linear regression with only one independent variable *b* is called simple linear regression, and the equation describing it is the following [63]:

(4) $$a=\beta_{0}+\beta_{1}\times b+\epsilon ,$$

where $\beta_{0}$ is the intercept, $\beta_{1}$ is the gradient or regression coefficient, and ϵ is the random error, normally distributed for simple linear regression models. In that case, the null hypothesis for $\beta_{1}$ comes from the *t*-test and is therefore the same whether *a* is the dependent or the independent variable. Regression analysis can provide various coefficients that can be further processed or used directly in order to evaluate the accuracy of the model. As the aim of this analysis is the calculation of the correlation between surface and invasive recordings, the coefficient of determination ($R^{2}$-adjusted) was recruited.

The conventional coefficient of determination ($R^{2}$) describes how well the data fit the regression model and is expressed as a percentage, where 0% indicates that the dependent variable is not related at all to the built model, while 100% shows an absolute concordance between the dependent variable and the model. $R^{2}$-adjusted additionally adjusts for the number of terms that are added to the model in a way that only the predictors that really affect the dependent variable are considered, thus resulting in a more unbiased result. $R^{2}$-adjusted is always equal to or less than $R^{2}$.

In general, cross-entropies allow the comparison between two time-series of different origins [64,65]. They are used to evaluate dynamic changes between two series and observe any similarities they may have. For this analysis, CQSE was chosen as an enhanced version of cross-sample entropy, allowing the tolerance *r* to vary in order to achieve better conditional probability estimates [66].

Any bias due to discrepancies in the values of the time-series should be removed before performing the CQSE [65]. This is achieved by normalizing each time-series as follows:

(5) $$x^{\prime }\left(i\right)=\frac{x\left(i\right)-\bar{x}}{std(x)},$$

where $x^{\prime }\left(i\right)$ is the *i*-th sample of *N*-length time-series X={X(1),X(2),…,X(N)}, $\bar{x}$ is the mean value, and std is the standard deviation.

Details about the computation of CQSE can be found elsewhere [65,66]. In brief, given two time-series X={X(1),X(2),⋯,X(N)} and Y={Y(1),Y(2),⋯,Y(M)} of length *N* and *M*, respectively, the probability that $X_{i}^{m}$ patterns of length *m* are similar to $Y_{j}^{m}$ patterns with a tolerance *r* is

(6) $$A^{m}\left(r\right)=\frac{1}{N-m}\times \sum_{i=1}^{N-m}\left[\;\frac{1}{M-m}\sum_{j=1}^{M-m}\times \Theta \left(r-d_{ij}^{m}\right)\right]\;,$$

where Θ is the Heaviside function and $d_{ij}^{m}$ the distance between $X_{i}^{m}$ and $Y_{j}^{m}$.

Respectively, for a template of length m+1, the probability that two patterns $X_{i}^{m+1}$ and $Y_{j}^{m+1}$ are similar is the following:

(7) $$A^{m+1}\left(r\right)=\frac{1}{N-m-1}\times \sum_{i=1}^{N-m-1}\left[\;\frac{1}{M-m-1}\sum_{j=1}^{M-m-1}\times \Theta \left(r-d_{ij}^{m+1}\right)\right]\;.$$

Then, CQSE is calculated as

(8) $$CQSE\left(m,r,N,M\right)=-ln\frac{A^{m+1}\left(r\right)}{A^{m}\left(r\right)}+ln\left(2r\right).$$

After multiple iterations, this analysis was performed with m=1 and r=0.35, as these parameters provided the best results.

## 3. Results

**Figure 2:**
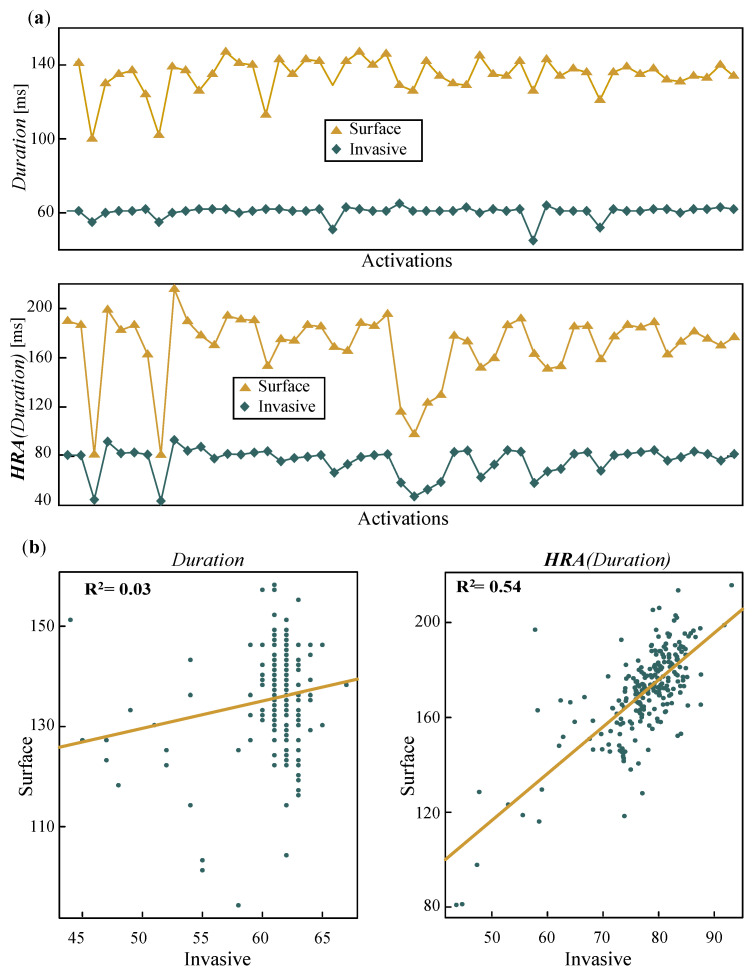
Example of how HRA affects the correlation of the computed features. (**a**) Time instance of 50 activations for the correlation of *Duration*, unprocessed (**top**) and after HRA (**bottom**). (**b**) Linear regression of the same recording before (**left**) and after (**right**) HRA. Linear correlation increased from $R^{2}=0.03$ to $R^{2}=0.54$. HRA: heart-rate adjustment.

The effect of scaling for HRA on *Duration* can be observed in Figure 2. While *Duration* values between each P-wave and CS LAW do not seem to correlate, when HRA is applied, they seem to follow a more similar pattern (Figure 2a). For the same recording, linear regression notably improves after HRA, and the values seem more coherent (Figure 2b).

### 3.1. Linear Analysis

**Figure 3:**
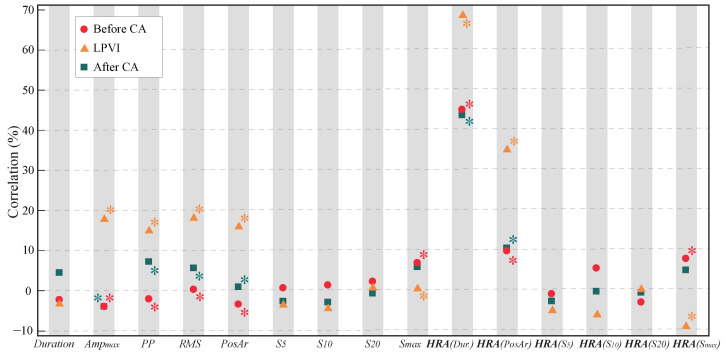
Pearson correlation between surface and invasive features of each activation, measured before CA (red), after LPVI or during CA (green), and after the end of the CA procedure (blue). Statistically significant results are marked with an asterisk (∗).

The results of Pearson correlation when each P-wave were compared with the corresponding LAW (P-wave/LAW analysis) by Pearson’s correlation, as illustrated in Figure 3 and shown further in detail in Table 1. *Amplitude* and *Area* features showed low positive and negative statistically significant correlations (−3.92% to +18.36%, p<0.0142) regardless of the observation point (recordings before, during, or after CA). $S_{max}$ also showed a negligent positive correlation in recordings before and during CA. No statistically significant correlations were found for the remaining features.

After HRA, HRA(Duration) showed a notably higher correlation (+43.82% to +69.91%, p<0.0001). The correlation between HRA(Area) of each P-wave and LAW also increased, still showing low values (+10.00% to +35.40%, p≤0.0425). The effect of scaling on $S_{max}$ was minor, with correlations either remaining almost the same or becoming negative (+0.71%,p=0.0114 for non-HRA versus −8.65%,p=0.0091 for HRA in recordings during CA).

When LR analysis was performed, statistical power remained the same for each feature as in Pearson correlation analysis. Nonetheless, correlations became weaker due to cross-validation. The effect of HRA was also present, converting *Duration* from low and statistically insignificant (+0.22% to +0.69%, p<0.2221) to low or moderate and statistically significant (+18.97% to +47.25%, p<0.0001) for HRA(Duration), as can be observed in Table 2. In both Pearson’s correlation and LR, features reached higher concordance during CA (after LPVI), especially after HRA.

**Figure 4:**
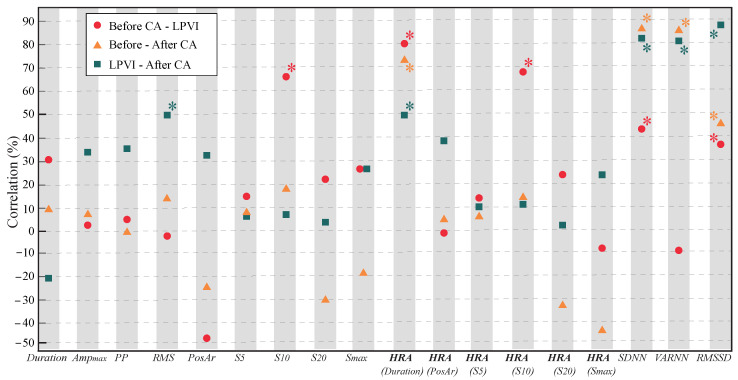
Pearson correlation between surface and invasive features for the variation measured between the recordings before CA and after LPVI (red), before the beginning and after the end of the CA procedure (orange), and after LPVI and after the end of the CA procedure (green). Statistically significant results are marked with asterisk (∗).

CV between P-waves and LAWs can be seen in Figure 4 and Table 3. Before HRA, only *RMS* (+54.07%,p=0.0205) at the third transition, measuring the difference in features between the end of LPVI and the end of CA and $S_{10}$ (+26.29%,p=0.0009) at the first transition, measuring the difference in features between the beginning of CA and the end of LPVI, showed small to moderate correlations. In this case as well, HRA enhanced this effect for $\mathrm{HRA}(S_{10})$ (+73.23%,p=0.0055) and additionally revealed a moderate to high CV for HRA(Duration) (+53.90% to +85.72%, p<0.0210). The effect of CA in ARV was quite similar for P-waves and LAWs, as can be observed from the moderate to high CV that they showed (+48.33% to +94.20%, p<0.0422). While the effect of LPVI seemed to affect in a more similar way the activation-based features and especially HRA(Duration) of P-waves and LAWs, CV for ARV features was notably lower after LPVI, lacking statistical significance in most of the cases.

### 3.2. Nonlinear Analysis

**Figure 5:**
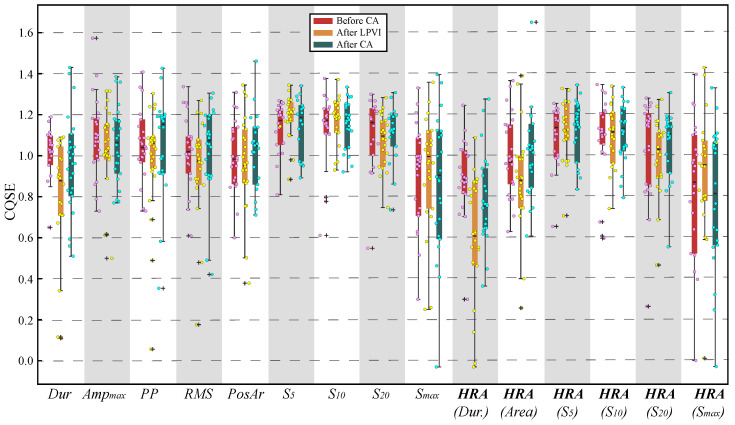
Combined box and whiskers plot with scatterplot for CQSE between surface and invasive features before (**left**), after LPVI (**centre**), and after full CA (**right**). *Dur*: *Duration*.

The scatterplot of Figure 5 shows median CQSE values for all features of each recording. From the box and whiskers plot of the same figure, it can be seen that the median values are found in the range from about 0.6 to 1.2, a fact that does not suggest strong nonlinear relationships between surface and invasive features. Unlike linear analysis, scaling for HR compensation did not improve the nonlinear relationships.

## 4. Discussion

Invasive EGM analysis is at the frontline of atrial substrate evaluation, assisting the detection of areas with structural remodeling [7,10]. During AF, these areas are specified as areas with a high dominant frequency and low-voltage EGMs or areas with highly complex EGMs, and various techniques have been developed to facilitate their detection as possible non-PV ablation targets [7,67,68]. During SR, the detection of areas sustaining the AF activity is achieved with the help of specific atrial sites that are used for pacing [10,14]. CS is the principal atrial site used for this purpose, allowing the detection of non-PV triggers spanning throughout the atria, while it has also been the object of non-PV CA, as it can trigger or sustain AF [14,16,22,23].

Considering the pivotal role of CS in AF perpetuation, as well as in AF mapping during CA procedures, more concrete knowledge on CS function and how it is affected by CA allows a different perspective on the atrial substrate modification due to the CA of AF. At the same time, given the popularity and effectiveness of P-waves and HRV in the atrial substrate modification analysis, correlating CS LAWs with P-waves acquired from ECGs allows a direct comparison with one of the most established methods in assessing the substrate modification. This was the original objective of the present study. For this purpose, recordings before, during, and after CA were acquired.

Before HRA, correlations found either between each P-wave and CS LAW or between lead II and CS recordings, when the correlation of CA-induced variations was assessed, were mainly loose and observed when *Amplitude*, *Area*, or *Slope rate* features were analyzed. Therefore, in SR, any attempt to observe the P-waves’ evolution through CS recordings or vice versa would lead to the loss of information. The effect of each CA step on P-waves and CS LAWs was not similar either. Nonlinear analysis also failed to detect any strong relationships between P-waves and CS LAWs. Despite the important role of CS in CA, the aforementioned findings suggest that it functions in a way that is different than that observed from the P-waves, which refer to the entire atria.

Although no significant correlations between P-wave and CS LAW features were detected, the present study led to an interesting revelation. Recordings after LPVI were acquired while RF energy was being emitted to PVs, possibly affecting the functionality of the atria. At the same time, RF energy was proven to affect the autonomous nervous system both during and after RFCA, in a different way [48,69,70]. During RF energy application, HR is decreased and HRV is increased. HRV incrementation implies a possible masking of the correlation in *Duration* between surface and invasive recordings, which should be unmasked after HRA. Indeed, although in all cases, HRA had a significant effect in the correlation of *Duration*, comparison after LPVI especially benefited from the application of HRA to the analyzed features. As CS is closer to the tissue under ablation, it is quite possible that HRV is more intense in CS recordings, being responsible for the low and negative correlations observed before HRA.

When assessing the correlation of the effect of each CA step between P-wave and CS LAW features, LPVI was once more the critical step that affected the variation of *Duration* in a more similar way. In our case, ARV was measured from time-domain HRV analysis on the atrial instead of ventricular activations of lead II and CS recordings in order to focus on the atrial rate and its fluctuations along the procedure. Unlike activation-based features, higher discrepancies between the ARV of surface and invasive recordings were observed after LPVI with respect to the remaining CA steps. This observation corroborates the theory of the different and probably more prominent effect of RF energy on CS function, manifested by the difference in ARV between the surface and invasive recordings during RF exposure and explains the significant role of HRA in potentiating the correlation of *Duration*. It should also be noted that although not highly correlated, the effect of CA on the *Duration* of P-wave and LAWs after HRA is similar. After the end of the procedure, ARV correlation between surface and invasive recordings notably increased. Previous studies reported temporary autonomous nervous system impairment as a consequence of RFCA manifested by decreased HRV [48,49]. This effect may also explain the high correlation of ARV features after the CA procedure in the present study.

As already mentioned, scaling in order to compensate for the HR variations had a major effect on linear analysis results regarding *Area* and *Duration* features. Moderate or moderate to high correlations were revealed for the latter, reaching up to 85.72% of concordance regarding the effect of LPVI on P-wave and CS LAW HRA(Duration). These correlations were hidden by the variable HR observed across the recordings due to the effect of RF applications [61,71]. Although correlation levels between P-wave and CS LAW *Duration* even after scaling do not imply an absolute tuning, it would be impossible to appreciate the similarity degree without this adjustment. As P-wave *Duration* is probably one of the most highlighted atrial electrical characteristics recruited to assess atrial substrate modification [33,36,41,42,72], the unbiased processing of this feature is of high importance. Therefore, studies combining the analysis of surface and invasive recordings acquired during SR are highly suggested to apply scaling techniques in order to achieve more robust results.

In AF research, the correlation between surface and invasive recordings has been primarily performed in order to verify the reliability of noninvasive methods in describing or mapping the atrial substrate [73]. Nonetheless, some of them report interesting findings regarding the association between parameters from ECG recordings and invasive EGMs. During SR, correlations have been found between the *Duration* of lead II P-waves and right atrium (RA) LAWs on AF and sick sinus syndrome patients [74]. Nonlinear correlations between RA recordings and recordings from lead II or V1 have also been observed during AF [75]. Finally, the analysis of post-operative recordings of V1 channel and unipolar RA EGMs during AF showed high correlations on *f*-wave organization and amplitude [76].

Compared to the present study, an essential difference exists in the aforementioned works. The invasive part of the these studies employed recordings from various RA sites, where fibrosis can also be found, versus recordings acquired only from CS in the present study [77]. Given the position of CS between the left atrium (LA), where PVs are found, and the RA, which also contributes to the AF perpetuation, and additionally taking into account the previous works, we would accordingly expect a more direct and robust relationship between CS LAWs and P-waves, which in any case was weak, but not negligent.

A limitation of the present study, which could affect the weak CS LAWs’ and P-waves’ correlations, is the not very large dataset analyzed. A significantly wider database could reaffirm or, by contrast, vary the findings of the current study. In any case, electrical substrate modification involves multiple atrial sites, expressing atrial alteration in a cumulative way. Therefore, one possible explanation, other than the small dataset, is that substrate alteration is a collective process, and the correlation between P-waves and various atrial sites, one by one, would fail to show significant relationships. Another possible explanation is that specific atrial sites correlate to a higher degree with P-wave behavior, and CS is not one of them. In that case, correlation with more atrial sites should be investigated. Nevertheless, this is a complex procedure, as the catheter placed in the atria is constantly moving and continuous recordings from one site cannot be easily acquired.

## 5. Conclusions

The lack of very high linear or nonlinear correlations between surface P-waves and invasive CS LAWs may discourage the employment of CS analysis to assess CA-induced changes of atrial function, as well as the possibility to predict the effect of CA from CS LAWs’ analysis. However, a scaling technique to mitigate the effect of variable HR has notably potentiated the surface-invasive correlations, and its implementation is suggested in analyses comparing between the characteristics of surface and invasive recordings. For a more detailed and in-depth analysis, the processing of simultaneous recordings both from surface ECG leads and CS EGMs is also encouraged.

## Figures and Tables

**Table 1:** Correlations (%) and *p* values for Pearson analysis between surface and invasive features for each and every activation. Statistically significant results are shown in bold. LPVI: left pulmonary vein isolation; *PP*: peak-to-peak amplitude; *RMS*: root mean square; *S_i_*: *Slope rate* at *i*% of *Duration*.

	Corr_pre_		Corr_LPVI_		Corr_post_	
Feature	*ρ* [%]	*p* Value	*ρ* [%]	*p* Value	*ρ* [%]	*p* Value
*Duration*	−2.19	0.1291	−3.08	0.1040	4.53	0.2221
$Amp_{max}$	−3.87	0.0001	18.05	0.0001	−3.92	0.0001
*PP*	−1.98	0.0090	15.19	0.0033	7.26	0.0016
*RMS*	0.38	0.0019	18.36	0.0017	5.46	0.0142
*PosAr*	−3.30	0.0032	16.18	0.0015	0.99	0.0142
$S_{5}$	0.76	0.4283	−3.93	0.1863	−2.59	0.2170
$S_{10}$	1.47	0.2359	−4.15	0.3062	−2.82	0.2923
$S_{20}$	2.38	0.0839	0.97	0.3927	−0.64	0.1426
$S_{max}$	7.05	0.0066	0.71	0.0114	5.96	0.1300
HRA(Duration)	45.25	<0.0001	69.91	<0.0001	43.82	<0.0001
HRA(PosAr)	10.00	0.0142	35.40	<0.0001	10.66	0.0425
$\mathrm{HRA}(S_{5})$	−0.70	0.2707	−4.84	0.0703	−2.55	0.2249
$\mathrm{HRA}(S_{10})$	5.71	0.2337	−5.68	0.1593	−0.13	0.1738
$\mathrm{HRA}(S_{20})$	−2.78	0.1431	0.60	0.0843	−0.44	0.1187
$\mathrm{HRA}(S_{max})$	8.09	<0.0001	−8.65	0.0091	5.21	0.1623

**Table 2:** Linear regression analysis results between surface and invasive features for each and every activation. Statistically significant results are shown in bold.

	Pre-CA		LPVI		Post-CA	
Feature	*R*^2^-adj [%]	*p* Value	*R*^2^-adj [%]	*p* Value	*R*^2^-adj [%]	*p* Value
*Duration*	0.56	0.1291	0.69	0.1040	0.22	0.2221
$Amp_{max}$	2.68	0.0100	4.27	0.0001	2.12	0.0094
*PP*	2.12	0.0090	2.34	0.0033	3.49	0.0016
*RMS*	5.33	0.0019	3.60	0.0017	4.12	0.0142
*PosAr*	2.94	0.0032	3.24	0.0015	1.40	0.0148
$S_{5}$	0.12	0.4283	0.32	0.1863	0.20	0.2170
$S_{10}$	0.17	0.2359	0.02	0.3062	0.05	0.2923
$S_{20}$	0.61	0.0840	0.09	0.3927	0.38	0.1426
$S_{max}$	2.07	0.0066	1.98	0.0114	0.52	0.1300
HRA(Duration)	20.32	<0.0001	47.25	<0.0001	18.97	<0.0001
HRA(PosAr)	1.90	0.0142	12.22	<0.0001	1.03	0.0425
$\mathrm{HRA}(S_{5})$	0.72	0.2707	0.86	0.0703	0.18	0.2249
$\mathrm{HRA}(S_{10})$	0.14	0.2337	0.43	0.1593	0.24	0.1739
$\mathrm{HRA}(S_{20})$	0.47	0.1431	0.78	0.0843	0.47	0.1187
$\mathrm{HRA}(S_{max})$	7.65	<0.0001	2.11	0.0091	0.38	0.1623

**Table 3:** Pearson’s correlations (%) and *p* values between LAWs and P-waves for CV measured every two ablation steps. Statistically significant results are shown in bold. CV: correlation of variation.

	CV_pre CA−LPVI_		CV_pre−post CA_		CV_LPVI−post_	
Feature	*ρ* [%]	*p* Value	*ρ* [%]	*p* Value	*ρ* [%]	*p* Value
*Duration*	34.82	0.1567	13.31	0.5984	−17.58	0.4854
$Amp_{max}$	6.16	0.8081	11.40	0.6253	38.24	0.1173
*PP*	8.67	0.7326	3.56	0.8885	39.20	0.1077
*RMS*	1.41	0.9556	18.28	0.4679	54.07	0.0205
*PosAr*	−43.37	0.0722	−20.56	0.4131	36.60	0.1352
$S_{5}$	18.78	0.4555	11.53	0.6486	10.31	0.6840
$S_{10}$	26.29	0.0009	22.54	0.3684	10.44	0.6803
$S_{20}$	26.29	0.2919	−26.10	0.2955	7.77	0.7593
$S_{max}$	−14.36	0.5690	−14.37	0.5695	31.11	0.2089
HRA(Duration)	85.72	<0.0001	78.89	<0.0001	53.90	0.0210
HRA(PosAr)	2.76	0.9135	9.01	0.7220	42.64	0.0776
$\mathrm{HRA}(S_{5})$	17.84	0.4788	10.33	0.6835	13.94	0.5811
$\mathrm{HRA}(S_{10})$	73.23	0.0055	18.91	0.4523	15.06	0.5508
$\mathrm{HRA}(S_{20})$	28.33	0.2546	−28.46	0.2523	5.88	0.8167
$\mathrm{HRA}(S_{max})$	−3.91	0.8776	−39.45	0.1052	28.70	0.2482
SDNN	48.33	0.0422	92.75	<0.0001	89.15	<0.0001
VARNN	17.44	0.4883	92.23	<0.0001	86.63	<0.0001
RMSSD	42.12	0.0817	51.23	0.0297	94.20	<0.0001

## Data Availability

The data supporting the reported results and presented in this study are available upon request from the corresponding author.
